# Correlations between lumbar neuromuscular function and pain, lumbar disability in patients with nonspecific low back pain: A cross-sectional study

**DOI:** 10.1097/MD.0000000000007991

**Published:** 2017-09-08

**Authors:** Haoyu Hu, Yili Zheng, Xueqiang Wang, Binglin Chen, Yulin Dong, Juan Zhang, Xiaochen Liu, Di Gong

**Affiliations:** a Department of Sport Rehabilitation, Shanghai University of Sport, Shanghai; b XuZhou Medical University, Jiangsu, China.

**Keywords:** lumbar disability, lumbar neuromuscular function, nonspecific low back pain, pain

## Abstract

This study aims to examine the correlations between lumbar neuromuscular function and pain, lumbar disability in patients with nonspecific low back pain (NSLBP).

Ninety patients, with ages 18 to 37 years old, with NSLBP were recruited in this study. The lumbar neuromuscular function was tested by the CON-TREX multijoint isokinetic test and training machine. This study uses the visual analog scale (VAS) and Roland–Morris Disability Questionnaire (RMDQ) to evaluate the pain and the dysfunction index of patients who have low back pain, respectively.

Pearson correlation coefficient is used to evaluate the correlation between lumbar neuromuscular function and the VAS and RMDQ scores. VAS and RMDQ scores have correlations with the proprioception in the flexion of the lumbar vertebra flexion; the peak torque of both flexion and extension muscle groups; and average power and endurance at different angular velocities. The decrease of lumbar muscle strength, endurance, and lumbar proprioception of the lumbar vertebra leads to an increase in pain intensity and lumbar disability.

This study suggests that patients with chronic low back pain require targeted training in muscle strength, endurance, and lumbar proprioception, providing a theoretical basis for prevention and treatment of chronic NSLBP patients.

## 1 Introduction

Low back pain is one of the most common symptoms with a lifetime prevalence rate of 84%.^[1]^ A pain that lasts for >12 weeks is defined as chronic low back pain,^[2]^ with 23% incidence rate.^[1,3]^ The main causes of low back pain are chronic strain, lumbar degeneration, bone hyperplasia, and disc herniation.^[1,3]^ Around 85% chronic low back pain has no specific diagnosis result or pathology, which is called “nonspecific low back pain” (NSLBP).^[2]^ NSLBP does not only affect health, quality of life, and work but also brings heavy medical burdens and indirect social costs.^[3]^ Therefore, NSLBP has become one of the major reasons of manpower loss and medical costs.^[4,5]^ In addition, patients with chronic low back pain experience a decrease in trunk activity because of the decrease in lumbar muscle strength and lumbar proprioception.^[6,7]^

The incidence rate of low back pain is high, and the daily lives of many patients have been affected.^[3,8]^ The neuromuscular function including several parts: muscle strength; muscle power; muscle endurance; voluntary muscle activation; and proprioception.^[9]^ The lumbar neuromuscular function may not only be responsible in improving the quality of patient's daily life and decreasing the pain, but also in maintaining the stability of the lumbar vertebra.^[10]^ Hence, the correlations of the lumbar neuromuscular function with pain and lumbar disability need to be explored. The lumbar neuromuscular function in this study includes 4 parts: lumbar proprioception, the strengths of lumbar flexion and extension muscle groups, average power, and endurance. The subjective evaluation index in this study is the visual analog scale (VAS),^[11]^ while the Roland–Morris disability questionnaire (RMDQ) allows the evaluation of the patient disability in daily life.

Previous studies^[12,13]^ revealed the correlation of VAS in low back pain cases with the muscle strength in flexion and extension muscle groups and the lumbar proprioception sense. These studies, however, cannot prove the correlations in lumbar disability. McGorry et al^[14]^ conducted the correlation of a longitudinal low back pain with function and suggested that pain–function correlations are stronger than those reported in cross-sectional studies over the course of low back pain. Most of these studies only show a unilateral correlation between lumbar neuromuscular function and pain and lumbar disability. According to the previous studies, we have found that the lumbar muscle strength and lumbar proprioception have a correlation with the VAS scores in low back pain cases. Moreover, in a longitudinal study, it has been proved that pain-function have a strong correlation even than cross-sectional studies. But few researches indicate both the correlation in pain and disability with lumbar neuromuscular function.

Therefore, the present study uses isokinetic muscle strength and lumbar proprioception test techniques to evaluate the neuromuscular function of the NSLBP crowd and to analyze the relationship between the lumbar muscle strength, endurance, lumbar proprioception capacity, pain, and lumbar disability. This paper's results may provide us new information and theoretical basis on better treatment and rehabilitation of chronic NSLBP patients.

## 2 Methods

### 2.1 Ethical considerations

Before the intervention, each subject will be asked to sign a written informed consent. The study was approved by the ethics committee of the Shanghai University of Sport, China.

### 2.2 Sample size estimation

Kovacs et al^[8]^ published the correlation between pain intensity in low back pain patients and lumbar disability in 2004 with the correlation index at 0.422. In the present study, we used G∗Power Software: *t*-test (version 3.1.9.2, FranzFaul, Universitat Kiel, Germany), at effect size, test level (α), test efficacy (1–β), and total sample size of 0.4, 0.05, 0.90, and at least 47, respectively.

### 2.3 2.2Study subjects

We chose the chronic low back pain patients who received the treatment in the orthopedic hospital of Shanghai Sports University from May 2014 to March 2015 and the chronic NSLBP students from the same institution as test subjects. A total of 90 subjects from 18 to 37 years old participated in the study. All subjects should perform the x-ray and MRI to exclude specific low back pain. And the medical doctor did the lumbar functional test such as: lumbar flexion, extension and rotation. The inclusion criteria set were: subjects aging 18 to 60 years old who could understand the language and volunteered to participate; stable vital signs, conscious, and without cognitive impairment; and the course of low back pain ≧3 months. The exclusion criteria set were: previous intellectual disabilities; severe heart, liver, kidney, lung disability, tumor, pregnant women, and postoperative disability; cardiovascular and cerebrovascular diseases; mental illness or long-term use of sedatives; musculoskeletal system diseases that disturb neuromuscular function (such as lumbar disc protrusion, lumbar fracture, severe arthritis, bone lumbar stenosis, ankylozing spondylitis, and so on). The doctor has excluded the specific low back pain patients by asking the history; performing the physical examination and doing the laboratory test.

### 2.4 Lumbar proprioception test

**Figure 1:**
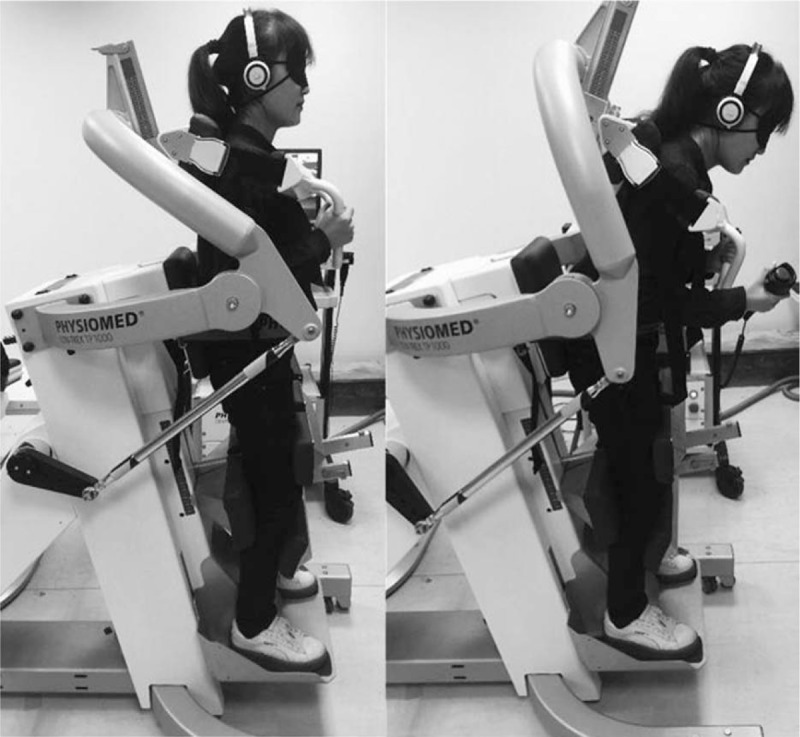
Lumbar proprioception test.

We used the CON-TREX multijoint isokinetic test and training machine (CMV AG, Dübendorf, Switzerland) for the lumbar proprioception test. The specific test methods are as follows: attempted removal of visual and auditory effects in the subject; slow trunk flexion of the subject from the original position to a predetermined target angle by a constant-velocity machine and maintaining that position for 3 seconds, and reminding the subject to remember this predetermined target before the machine returns the trunk to the original position; and trunk movement with the subject holding the remote control and pressing the pause button at the target angle, which is recorded as the actual angle. The difference between the actual angle and the target angle is the absolute error angle, which is used to assess the ability of position sense. The test is measured in triplicates, and the absolute error angle is taken as an average. In addition, subjects can practice 3 times to adapt to this isokinetic test machine before conducting the formal proprioceptive test. The picture is shown in Fig. 1.

### 2.5 Isokinetic muscle strength test of the lumbar

The isokinetic muscle strength test of the lumbar is performed using the CON-TREX multijoint isokinetic test and training machine (CMV AG, Dübendorf, Switzerland). The test methods are as follows: The subjects take an upright position and fasten the shoulder blade and the pelvis with a drawstring. The power instrument axis aligns with the subject's trunk on L5–S1. The shoulder is fixed to the scapula, the hip joint is fixed to the pelvis, and the knee slightly bent and fixed above and below the feet placed on the adjustable pedals. The subject can familiarize with the process. The angular speed can be selected as 90°/s, and maximum contraction was performed 5 times as a warm-up exercise. Next, angular speed is selected as 60°/s, 120°/s, and 180°/s to begin the formal isokinetic strength test. Using centripetal–centripetal contraction at constant velocity, the subjects can individually use their best strength to flex and extend the lumbar 10 times at 3 different angular velocities. Each section rests ∼90 seconds. The picture is the same illustration shown in Fig. 1. The isokinetic muscle strength test provides several indicators, such as flexion and extension muscle peak torque, the average power of the lumbar vertebra flexion and extension, the endurance of the lumbar vertebra flexion and extension muscle. General information on the neuromuscular function indicators of the subject are listed in Table 1.

**Table 1:**
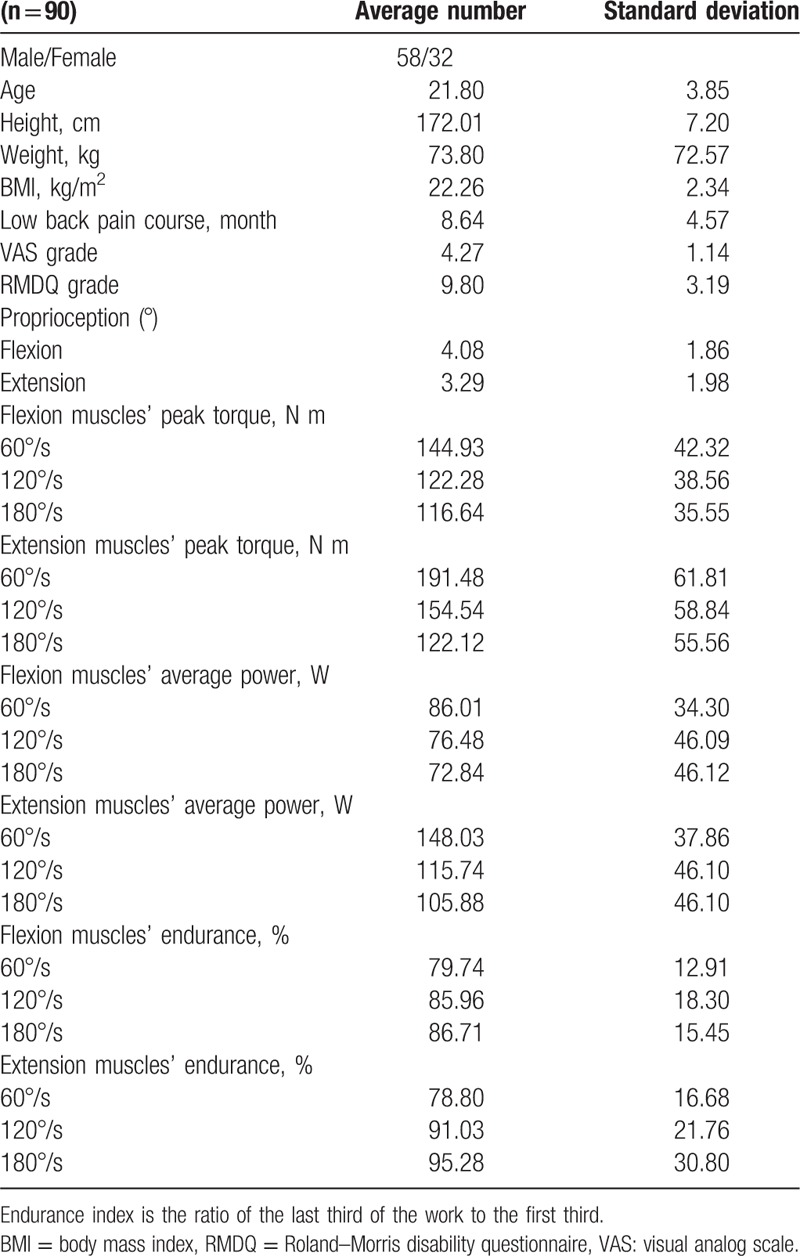
General information on subjects and neuromuscular function indicators.

### 2.6 Assessment of pain

**Figure 2:**
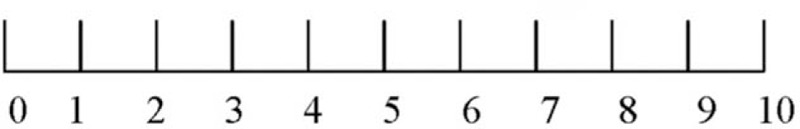
Visual analog score.

The VAS^[11]^ evaluates pain intensity in patients with low back pain. VAS scores are measured on a 10-cm horizontal line, with “0,” “1–4,” “5–6,” “7–9,” and “10” indicating “no pain,” “mild pain,” “moderate pain,” “severe pain,” and “unbearable pain,” respectively.^[11,15]^ The patients point out the score in the horizontal line. The picture is shown in Fig. 2.

### 2.7 Assessment of lumbar disability

RMDQ was used to evaluate the dysfunction index of patients with low back pain. The questionnaire was designed by Roland and Morris to mainly reflect the overall health of patients who have low back pain.^[16]^ The RMDQ has 24 yes–no questions. A “yes” answer is equivalent to 1 point, while “no” means 0 point. Out of the total score of 24 points, the higher the acquired point indicates a more serious problem in lumbar vertebra disability. Both scholars Fan and Yi^[17,18]^ did the research in the reliability and validity on SCRMDQ (simplified Chinese version of RMDQ). The result showed that the Cronbach α value for internal consistency is 0.826 in Fan et al's paper and 0.874–0.883 in Yi et al's paper. The intraclass correlation coefficient (ICC) value is 0.949–0.952 in Yi et al's paper and 0.947 in Fan et al's paper. So the SCRMDQ has a good reliability and validity. In this research, we use SCRMDQ to evaluate the dysfunction index of patients with low back pain.

### 2.8 Statistical analysis

Collected data were submitted to SPSS 17.0 and Microsoft Excel 2007 for analysis. The data is presented as “average ± standard deviation.” Pearson correlation coefficient was used to evaluate the correlation between lumbar muscles strength, endurance, proprioception, and the VAS and RMDQ scores. The significance level was *P* <.05. The higher absolute value of Pearson correlation coefficient indicates a stronger correlation.^[19,20]^ Scores of 0.0–0.2, 0.2–0.4, 0.4–0.6, 0.6–0.8, and 0.8–1.0 points represent “extremely weak or no correlation,” “weak correlation,” “moderate correlation,” “strong correlation,” and “extremely strong correlation,” respectively.

## 3 Results

The correlation between pain, disability, and proprioception in chronic NSLBP patients is shown in Table 2. VAS scores are correlated with the proprioception in lumbar vertebra flexion (*r* = 0.268, *P* = .011). RMDQ scores are correlated with the proprioception in lumbar vertebra flexion (*r* = 0.317, *P* = .002) and extension (*r* = 0.218, *P* = .039).

**Table 2:**
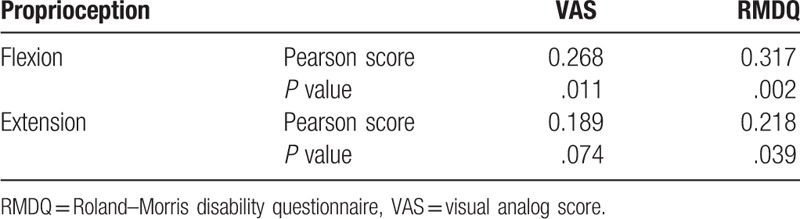
Correlation between pain, disability, and proprioception (n = 90).

The correlation between pain, disability with lumbar flexion, and extension peak torque in chronic NSLBP patients is shown in Table 3. VAS scores are correlated with flexion muscle at 60°/s (*r* = −0.504, *P* <.001) and with extension muscle at 60°/s (*r* = −0.389, *P* <.001), 120°/s (*r* = −0.301, *P* = .004), and 180°/s (*r* = −0.429, *P* <.001). RMDQ scores are correlated with flexion muscle at 60°/s (*r* = −0.503, *P* <.001) and with extension muscle at 60°/s (*r* = −0.341, *P* <.001), 120°/s (*r* = −0.295, *P* = .005), and 180°/s (*r* = −0.374, *P* <.001).

**Table 3:**
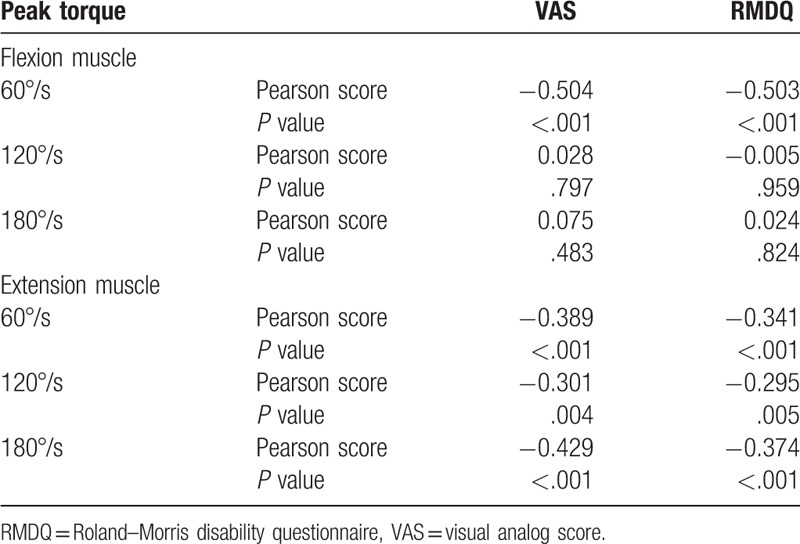
Correlation between pain, disability and peak torque (n = 90).

The correlation between the pain and disability with the average power of the lumbar vertebra flexion and extension muscle in chronic NSLBP patients is shown in Table 4. VAS values show a correlation with average power in flexion muscle at 60°/s (*r* = −0.257, *P* = .014) and 120°/s (*r* = −0.423, *P* <.001) and with average power in extension muscle at 60°/s (*r* = −0.222, *P* = .036). RMDQ scores have a correlation with average power in flexion muscle at 60°/s (*r* = −0.249, *P* = .018) and 120°/s (*r* = −0.39, *P* <.01) and with average power in extension muscle at 60°/s (*r* = −0.226, *P* = .032).

**Table 4:**
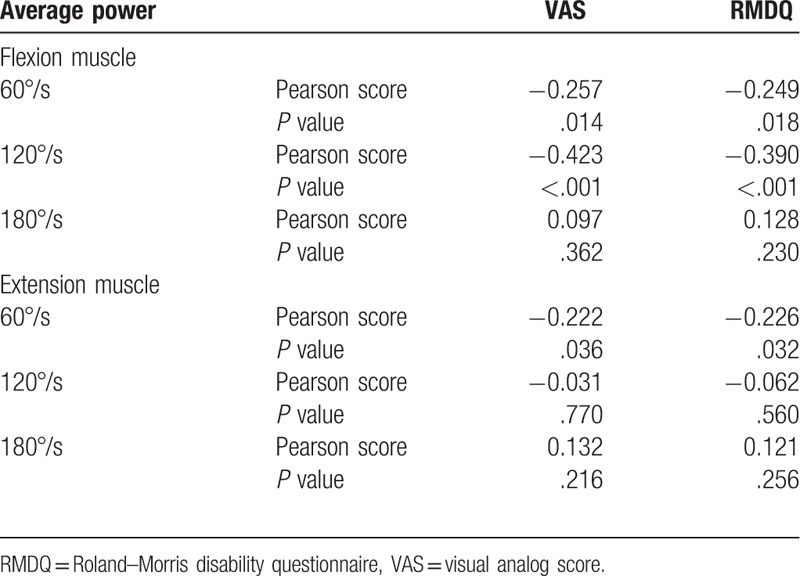
Correlation between pain, disability, and the average power (n = 90).

The correlation between the pain and disability with the endurance of the lumbar vertebra flexion and extension muscle in chronic NSLBP patients is shown in Table 5. VAS values show correlation with flexion muscle at 60°/s (*r* = −0.88, *P* <.001), 120°/s (*r* = −0.301, *P* = .005), and 180°/s (*r* = −0.511, *P* <.001) and with extension muscle at 60°/s (*r* = −0.376, *P* <.001). RMDQ scores show correlation with flexion muscle at 60°/s (*r* = −0.695, *P* <.001) and 180°/s (*r* = −0.369, *P* <.001) and with extension muscle at 60°/s (*r* = −0.358, *P* <.001).

**Table 5:**
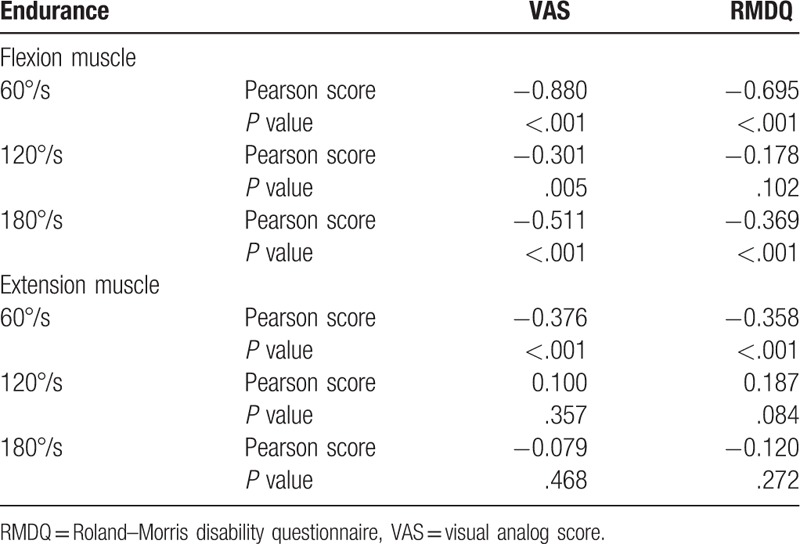
Correlation among pain, disability, and the endurance of the muscle (n = 90).

## 4 Discussion

### 4.1 Correlation among pain, disability, and proprioception

This study selects the joint location as an indicator of proprioception. The current test method for proprioception is the joint position test. The results indicated that the lumbar proprioception has correlations with pain and lumbar disability. Roosink et al^[21]^ observed that pain intensity in 15 patients with chronic low back pain is correlated with proprioception sense and the worsening degree of proprioception from symptom aggravation. According to our research and Roosink's paper, the pain may decrease the sensitivity of the proprioceptive receptor so the NSLBP patients’ lumbar proprioception may worse than the normal persons. Conrad et al^[6]^ also found that the Oswestry disability index (ODI) in lumbar stenosis patients weakly correlate with the proprioception sense (*r* = 0.213, *P* = .036). Therefore, simulation of the proprioceptor and improvement of the proprioception sense are necessary to decrease the pain and improve the lumbar function in patients with low back pain.

### 4.2 Correlation among pain, disability, and peak torque

This study selects peak torque as an indicator for muscle strength. The isokinetic muscle strength test for peak torque is the “gold standard” method for evaluating muscle strength because of its high repeatability and accuracy. Guilhem et al^[12]^ have shown the good reliability of measuring peak torque in body isokinetic muscle strength test (ICC = 0.87–0.95). In this research, the peak torque has correlation with both lumbar pain and disability. These results confirmed the deeper pain intensity and the higher disability index in patients with chronic low back pain. When the lumbar and abdomen muscle weakens, lumbar stability decreases, and the symptoms of the lumbar worsen. Verbunt et al^[22]^ found that the pain intensity of 25 patients with chronic low back pain results to poor muscle activation ability. Therefore, training the shallow and deep core muscle is particularly important for patients with chronic low back pain.

### 4.3 Correlation among pain, disability, and average power: muscle endurance

The average power is a reflection of the work efficiency, and it is a common indicator for evaluating isokinetic muscle strength. Santos et al^[23]^ confirmed the reliability of the average power indicator. Iwai et al^[24]^ evaluated the significant correlation between lumbar disability and average power in 53 patients with low back pain (*r* = −0.49, *P* <.05). In the isokinetic muscle strength assessment, the endurance index is the ratio of the last third of the work to the first third, where a higher ratio indicates higher fatigue resistance and better muscle endurance.^[13]^ Based on the VAS scores, ODI scores, lumbar muscle strength, and muscle endurance, the pain intensity, lumbar disability, and lumbar muscle endurance improved after 6-week suspension training of the chronic low back patients.^[25]^ These results indirectly show the correlation of pain intensity and lumbar disability with the lumbar muscle endurance in patients with chronic low back pain. Snekkevik et al^[26]^ established the correlation of fatigue with the intensities of pain and lumbar disability in 569 chronic low back pain patients, where the deeper intensity of lumbar disability indicates higher fatigue.

## 5 Limitations

This study is limited on the following disadvantages: The low back pain subjects are all young people with an average age of 23.98 years old. The age is between 20 and 34 years. Hence, the result cannot represent the entire human population. Rather than including the proprioception senses of motion perception and vibration perception, we only tested the position perception as motion and vibration perceptions are difficult to quantify. Hence, an accurate and comprehensive reflection of proprioception function is necessary and requires improvement as an important research direction. Muscle endurance reflects neuromuscular function. Further exploration of a better index replacement is necessary.

## 6 Clinical significance

This study finds that the proprioception has correlations with pain and function in NSLBP patients. Hence, if we use the CON-TREX multijoint isokinetic test and training machine to do the lumbar proprioception training, low back pain patients may activate more proprioceptors to improve the proprioception.

Both lumbar muscles strength and endurance have a correlation with pain and function in different flexion and extension angles. If we add the lumbar muscle strength and endurance training during the rehabilitation, the lumbar pain and dysfunction may decrease a lot. According to the results of the research we should do more training on muscle strength in flexion and endurance in extension.

## 7 Conclusion

This project uses the proprioception, isokinetic muscle strength, VAS, and RMDQ to evaluate the correlation among pain intensity, lumbar disability, lumbar muscle strength, proprioception, and muscle endurance in chronic NSLBP patients. The results suggest that patients suffering from chronic low back pain require targeted training in muscle strength, endurance, and lumbar proprioception. This study provides a theoretical basis for prevention and treatment of chronic NSLBP
